# Shorter Chain Triglycerides Are Negatively Associated with Symptom Improvement in Schizophrenia

**DOI:** 10.3390/biom11050720

**Published:** 2021-05-11

**Authors:** Anna Tkachev, Elena Stekolshchikova, Nickolay Anikanov, Svetlana Zozulya, Aleksandra Barkhatova, Tatiana Klyushnik, Daria Petrova

**Affiliations:** 1 V. Zelman Center for Neurobiology and Brain Restoration, Skolkovo Institute of Science and Technology, 121205 Moscow, Russia; E.Stekolschikova@skoltech.ru (E.S.); N.Anikanov@skoltech.ru (N.A.); D.Petrova@skoltech.ru (D.P.); 2 Mental Health Research Center, 115522 Moscow, Russia; s.ermakova@mail.ru (S.Z.); abarkhatova@yandex.ru (A.B.); klushnik2004@mail.ru (T.K.)

**Keywords:** schizophrenia, lipid, lipidomics, triglyceride, mass spectrometry, PANSS

## Abstract

Schizophrenia is a serious mental disorder requiring lifelong treatment. While medications are available that are effective in treating some patients, individual treatment responses can vary, with some patients exhibiting resistance to one or multiple drugs. Currently, little is known about the causes of the difference in treatment response observed among individuals with schizophrenia, and satisfactory markers of poor response are not available for clinical practice. Here, we studied the changes in the levels of 322 blood plasma lipids between two time points assessed in 92 individuals diagnosed with schizophrenia during their inpatient treatment and their association with the extent of symptom improvement. We found 20 triglyceride species increased in individuals with the least improvement in Positive and Negative Syndrome Scale (PANSS) scores, but not in those with the largest reduction in PANSS scores. These triglyceride species were distinct from the rest of the triglyceride species present in blood plasma. They contained a relatively low number of carbons in their fatty acid residues and were relatively low in abundance compared to the principal triglyceride species of blood plasma.

## 1. Introduction

Psychiatric disorders, including schizophrenia (SCZ), can be debilitating disorders that, if left untreated, give rise to severe problems that affect every area of life. While antipsychotics, among other possible additional treatments, are typically used as medication aimed to reduce symptoms of schizophrenia, treatment efficacy varies between individuals [1], and it is estimated that up to 34% of SCZ patients exhibit resistance to two or more antipsychotic medications [2]. Satisfactory biomarkers of treatment response and treatment resistance in schizophrenia would be extremely valuable, but are yet to be discovered [3]. Such biomarkers would not only be promising for personalized treatment plans, but also, possibly, provide fundamental information on the pathophysiology of the disorder.

Lipidomics in psychiatric disorders is an area of research gaining traction during the past years. In part, this can be attributed to the fast-developing field of lipid research [4,5]. There is also a growing awareness of the important function lipids are now known to play in both the properties and functionality of the brain, such as membrane fluidity and permeability, retrograde signaling, neural plasticity, and neurotransmitter release modulation [6,7]. Studies of blood plasma lipid alteration in psychiatric disorders, in particular, have been numerous over the past decade [8,9,10,11,12,13,14,15,16,17,18,19,20,21,22,23], with blood being a relatively easy sample type to procure for studies and most relevant for potential future biomarker implementations. Indeed, this area of research shows promise, evidenced by the distinct pattern of schizophrenia-associated lipid alterations being reported in the literature. Among other lipid compounds, the lipid classes most robustly associated with the disorder are ether phospholipids (such as plasmanyl-/plasmenyl- phosphatidylcholine and plasmanyl-/plasmenyl- phosphatidylethanolamine) [9,11,12,13,14,24] and acylcarnitines [8,10,15,25,26]. Associations with ether phospholipids and acylcarnitines have also been reported for other psychiatric disorders, such as major depressive disorder [19,20,21,27]. Additionally, ceramides have been proposed as a potential biomarker of depression [28].

While blood lipid alterations have been shown for psychiatric disorders, their levels are subject to a degree of variability related to sex, age, metabolic health, and, possibly, other unaccounted factors [29,30]. On the other hand, the study of changes over time in lipid levels of the same individual has the advantage of reducing this variability. Previously, several studies have aimed to investigate the association between changes in blood lipidome composition and symptom improvement for schizophrenia [31,32,33], but the available evidence is limited. In this study, we measured the blood lipid abundances in 92 individuals diagnosed with schizophrenia and undergoing inpatient treatment. The blood plasma was collected at two distinct time points to investigate the associations between symptom improvement and individual changes in blood plasma lipid levels.

## 2. Materials and Methods

### 2.1. Patients and Sample Collection

Included subjects were inpatients recruited from the Mental Health Research Center, Moscow. The subjects were adult (age range 17–43 years) participants with a diagnosis of SCZ (*n* = 92; 58% female) according to the ICD-10 criteria. Sample collection was performed at the Neuroimmunology Laboratory of the Mental Health Research Center, Moscow. Patient evaluation was performed by board-certified psychiatrists of the same center. Clinical symptoms of patients were assessed by Positive and Negative Syndrome Scale (PANSS) [34], a widely used medical scale of symptom severity in schizophrenia. The PANSS score is composed of three components: the positive (e.g., hallucinations and delusions), negative (represented by loss of normal functions), and general psychopathology components. Higher PANSS score values correspond to more severe symptom manifestations. The study was approved by the local ethics committee of the Mental Health Research Centre. Informed consent was obtained from all participants. The entire study was conducted in line with the World Medical Association Declaration of Helsinki formulating ethical principles for medical research involving human subjects.

Plasma was obtained from peripheral venous blood in the morning from individuals that underwent an overnight fast. Plasma samples were collected in 4 mL Vacutainer tubes containing the chelating agent ethylenediaminetetraacetic acid (EDTA) (BD Vacutainer, Franklin Lakes, NJ, USA). Tubes were centrifuged at 4 °C at 1100× *g* for 15 min. The supernatant was stored immediately in 500 μL aliquots at −80 °C.

### 2.2. Lipid Extraction and Lipidomics Measurements

A mixture of isotopically labeled lipids (SPLASH LIPIDOMIX Mass Spec Standard, Avanti Polar Lipids) was added to methyl tert-butyl ether (MTBE) based on 1 vial for 100 mL of solvent. All tubes and solvents were pre-cooled down to 0 °C and manipulations carried out on ice. Frozen plasma was thawed on ice for 2 h. Then, cold methanol (300 μL) was added to 40 μL of sample aliquot and vigorously vortexed for 1 min. Afterward, 1 mL of cold MTBE with spiked standards was added, and the mixture was sonicated for 10 min, incubated for 40 min at 4 °C in a shaker, and then sonicated for another 10 min. Phase separation was induced by adding 250 μL of MS-grade water. Extract was vortexed for 1 min at 4 °C, then centrifuged for 10 min at 13,000 rpm and 4 °C. A total of 1000 μL of the upper layer, containing most of the lipids, was collected in a fresh Eppendorf tube. Four hundred microliters of re-extraction buffer (MeOH:MTBE:H_2_O = 3:10:2.5) was added to the lower phase. Samples were vortexed and centrifuged for 10 min at 13,000 rpm and 4 °C. Additional 300 μL of upper phase was collected, and combined organic phases were evaporated to dryness in a Speed Vac concentrator at 30 °C. Dried lipid pellets were stored at −80 °C before analysis. The pellets were resuspended in 200 µL ice-cold acetonitrile–isopropanol mixture (7:3 (*v*:*v*)). After brief vigorous vortexing, the samples were shaken for 10 min, sonicated in an ice-cooled sonication bath for 10 min, and centrifuged 5 min at 13,000 rpm. For mass spectrometry analysis, samples were diluted 1:5 and 1:2 with acetonitrile–isopropanol (7:3 (*v*:*v*)) for positive and negative ionization modes, respectively.

The liquid chromatography/mass spectrometry system consisted of a Waters Acquity UPLC system (Waters, Manchester, UK) and a Q Exactive orbitrap mass spectrometer (Thermo Fisher Scientific, USA) equipped with a heated electrospray ionization (HESI) probe. Separation of lipids was performed at 60 °C using a reverse-phase ACQUITY UPLC BEH C8 Column (2.1 × 100 mm, 1.7 μm, Waters co., Milford, MA, USA) coupled to Vanguard precolumn at a flow rate of 0.4 mL/min. The mobile phases consisted of water containing 10 mM ammonium acetate (Buffer A), and a mixture of acetonitrile and isopropanol (7:3) containing 10 mM ammonium acetate (Buffer B). Both buffers contained 0.1% formic (positive mode) or acetic acid (negative mode) by volume. Separation was carried out by gradient elution according to the following profile: 1 min 55% B, 3 min linear gradient from 55% to 80% B, 8 min linear gradient from 80% B to 85% B, and 3 min linear gradient from 85% B to 100% B. After 4.5 min washing with 100% B, the column was re-equilibrated with 55% B for 4.5 min. The injection volume was 3 μL. Mass spectra were acquired both in positive and negative modes in different experiments with a mass range of *m*/*z* 100–1500 at a mass resolving power of 70,000 at *m*/*z* 200, AGC target: 1E6, Maximum IT: 100 ms. Ion source for scan mode was operated with following parameters: capillary temperature, 250 °C; aux gas heater temperature, 350 °C; spray voltage, 4.5 kV; S-lens RF level, 70; sheath gas (N_2_) flow rate, 45 arbitrary units (a.u.); auxiliary gas (N_2_) flow rate, 20 a.u.; sweep gas (N_2_) flow rate, 4 a.u. Data were acquired on the profile mode. External mass calibration (Pierce LTQ Velos ESI Positive Ion Calibration Solution, Pierce Negative Ion Calibration Solution) without the use of the specific lock masses was employed.

Additional experimental samples were analyzed together with the plasma samples. Empty tubes without plasma (extractions blanks) were placed in the end of the experiment and subjected to the same analysis steps as plasma samples. Quality control (QC) samples were incorporated every 12th position to account for sample preparation and measurement variability. QC samples consisted of aliquots of plasma, mixed before extraction.

For lipid structure elucidation, mass spectra were acquired in data-dependent acquisition mode with active inclusion list. Parameters for full MS scan mode were set as follows: resolution: 70,000 at *m*/*z* 200; AGC target: 5E5, IT: 50 ms, mass range: 200–1800 for both polarities. All ions from the inclusion list within 10 ppm range were subjected to fragmentation with the following parameters: AGC target: 5E4; IT: 100 ms. Intensity threshold was kept at 8E2 and isolation width was set at 1.2 Da. Stepped normalized collision energy was set at 15%, 20%, 25%, and 30%. Data were acquired on the profile mode, peptide match option was off, and isotope exclusion was set to on. Acquired spectra were manually curated and identifications were based on MS2 spectra, with MS1 mass error of <5 ppm and MS2 mass error of <8 ppm.

### 2.3. Lipidomics Data Processing

Spectra were processed using XCMS software [35], using the “centWave” method for peak detection, the “obiwarp” method for retention time correction, and the “fill-Peaks” method for missing value imputation. Duplicated features were deleted, and isotopes were removed as well, using an in-house algorithm. Abundance values were log2 transformed. Missing values as reported by XCMS were replaced by random values sampled from a normal distribution with mean 11.7 and standard deviation 0.25, which corresponds to the approximate noise level seen in the experiment. Batch effect was corrected for all samples, including QC and blank samples, by subtracting the median batch value, calculated excluding the QC and blank samples. The feature abundances were then returned to their original scale by adding the median value of abundances in each batch. Features retaining high technical variability after batch correction were removed using QC samples according to the following rule: features with standard deviation across QC samples > 1 (in log2 scale) were removed from the analysis. Contaminants were filtered out using extraction blank samples according to the following rule: features for which mean abundance in plasma samples < mean abundance in blank samples+1 (in log2 scale) were removed from the analysis. Features were also removed according to retention time thresholds 0 < RT < 19 for positive ionization mode and 0 < RT < 14.5 for negative ionization mode. Samples were not normalized according to total lipid content.

Lipid features were annotated by mass-to-charge (*m*/*z*) values and retention times with one select adduct per lipid class and *m*/*z* threshold of 10 ppm. Appropriate retention times were determined based on in-house retention time for previously annotated lipid species, as well as chain length and double bond content of the annotated lipid species. Additional MS2 experiments were performed to validate the annotation for part of the lipid compounds.

For downstream statistical analysis, only annotated lipid compounds were used.

### 2.4. Statistical Analysis

Wilcoxon signed-rank and Mann–Whitney U tests were used with continuity correction. 

When restricting patients to those treated with certain medications, all individuals that were administered this medication, among possible other drugs, were used, resulting in overlapping groups of patients.

Median base 2 log-transformed fold-changes (log2 fold-change) between the two time points were calculated as the median values of the individuals’ differences in lipid abundances between the second and first time points.

When calculating *p*-values for binomials test for correlation coefficients, the probability of a positive correlation was assumed to be ½.

When calculating *p*-values for binomial tests for median log2 fold-change for the subsets of worst and best responders, the probability of the median log2 fold-change of the subset of worst responders being larger than 0 was assumed to be ½. Likewise, the probability of the median log2 fold-change of the subset of worst responders being larger than the median log2 fold-change of the subset of best responders was assumed to be ½.

For analysis of variance (ANOVA) and interaction effect on lipid changes between time points, the following ANOVA type I model was used: lipid change ~ sex + age + PANSS change + sex * age + PANSS change * sex + PANSS change * age + PANSS change * sex * age. For ANOVA and interaction effect on lipid abundances at the first time point, the same model was used: lipid abundance at first point ~ sex + age + PANSS change + sex * age + PANSS change * sex + PANSS change * age + PANSS change * sex * age. To calculate the excess of nominally significant (*p* < 0.05) lipids for the different main effects and interactions, permutation test was performed. Sample labels were permutated 100 times for the lipid abundances table, and the number of nominally significant (*p* < 0.05) lipids for the different main effects and interactions was calculated each time. The fraction of permutations for which the random number was equal to or higher than the true number of nominally significant lipids was defined as the permutation *p*-value. The ratio of the median number of nominally significant lipids in the permutations to the true number of nominally significant lipids was used to define the fold excess of nominally significant lipids. Permutation test was used to calculate the enrichment of lipid classes among nominally significant lipids as well. A subsample of lipids of the same size as the number of nominally significant lipids was chosen at random, and the number of lipids from the given lipid class was calculated. The fraction of permutations (from 10,000) for which this random number was equal to or higher than the true number of nominally significant lipids from the given lipid class was used to define the permutation *p*-value.

## 3. Results

### 3.1. Study Setup

We assessed the abundance of 322 lipid species in the blood plasma of 92 individuals diagnosed with schizophrenia collected at two time points: at the beginning and end of their hospitalization at a psychiatric clinic (37 ± 19 days; Figure S1). Samples were represented by female and male individuals (58% female) of age ranges 17–43 years, and information on medication regiment was collected (Table S1). Symptom severity was assessed by Positive and Negative Syndrome Scale (PANSS) [34] score at the two time points (Table S1). Lipidomics measurements were produced using mass spectrometry coupled with liquid chromatography in negative and positive ionization modes. From the reproducibly quantified lipid features, a set of 322 unique lipid compounds was annotated based on their mass-to-charge, retention time values, and fragmentation patterns (Table S2). Assessed lipid species covered 14 lipid classes and aligned well with expected blood plasma lipidome composition [36] (Figure S2).

### 3.2. Association between Changes in Lipid Abundances and Symptom Improvement

**Figure 1:**
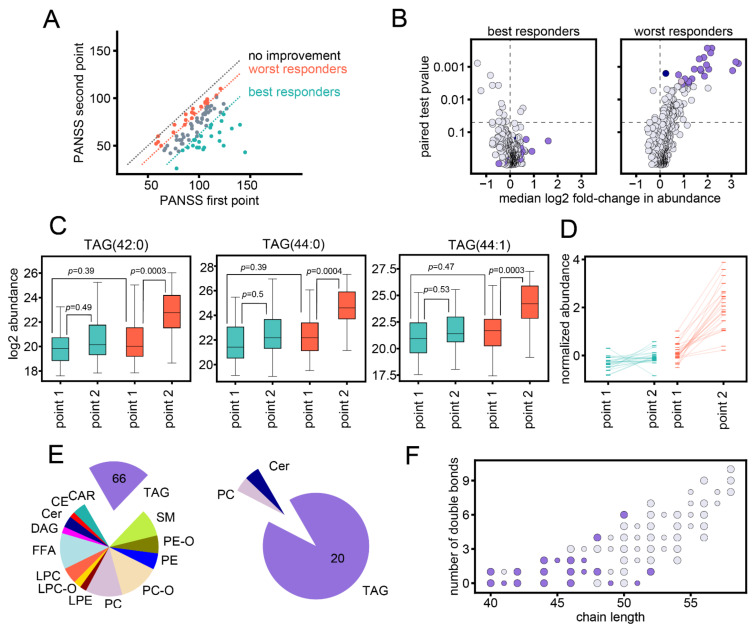
Significant changes in worst responders. (**A**) PANSS scores at first and second time points for the 92 individuals. Individuals with least improvement in PANSS score (worst responders, *n* = 24) and most improvement in PANSS score (best responders, *n* = 23) are marked in orange and green, respectively. Orange and green dashed lines demarcate to the upper and lower quartile of PANSS differences; dashed grey line corresponds to identical PANSS values at the two points. (**B**) *p*-values of the Wilcoxon signed-rank test plotted against the median base 2 log-transformed fold change (log2 fold-change) between the two time points for individuals with most improvement (left) and least improvement (right). The 22 worst-response-associated lipids are marked in color according to lipid class: TAG, purple; PC, dusty pink; Cer, dark blue. Dashed lines demarcate log2FC = 0 and nominal *p* = 0.05. (**C**) Log2 abundances for best and worst responders at first and second time points. Three worst-response-associated lipids with strongest statistical effect (*p* < 0.0005 for Wilcoxon signed-tank test for changes in worst responders) are plotted: TAG 42:0, TAG 44:0, TAG 44:1. Noted *p*-values correspond to Wilcoxon signed-rank and Mann–Whitney U test *p*-values for comparisons between groups. Boxplot whiskers and fliers correspond to standard boxplot definition. (**D**) The median values of the normalized log2 abundances for best and worst responders at first and second time points, for all 22 worst-response-associated lipids. For each lipid, the log2 abundances were normalized by the lipid mean value for all patients at the first time point. (**E**) Left: the 322 annotated lipids, grouped by lipid class. Right: the 22 worst-response-associated lipids, grouped by lipid class. The numbers of respective triglyceride species are indicated on the plot. (**F**) The number of carbons in the fatty acid residues (chain length) and number of double bonds for the annotated triglycerides. Worst-response-associated lipids are colored in purple. Larger and smaller circle sizes correspond to even and odd chain triglycerides, respectively.

All but one patient displayed symptom improvement from the first to the second time point, demonstrated by the reduction in PANSS scores (Figure 1A). However, the improvement in PANSS scores ranged from −102 to 0 point differences, with the top 25% best responders displaying PANSS score improvements of −102 to −39 point differences (*n* = 23) and the bottom 25% worst responders displaying PANSS score improvements of −14 to 0 point differences (*n* = 24) (Figure 1A). The extent of changes in lipid abundances differed depending on PANSS score improvement. While for worst responders, 22 lipids showed significant changes from first to the second time point (worst-response-associated lipids; Wilcoxon signed-rank test, Benjamini–Hochberg correction FDR 5%; Figure 1B; Table S2), the effect in best responders was lower (Wilcoxon signed-rank test, no significant lipids at Benjamini–Hochberg FDR 5% threshold; Figure 1B; Table S2). Accordingly, although the levels of worst-response-associated lipids at baseline were similar for best and worst responders, best responders did not display a statistically significant increase from first to the second point (respectively: Mann–Whitney U test, 1 of 22 *p* < 0.05; Wilcoxon signed-rank test, all 22 *p* > 0.05; Figure 1C,D; Table S2). Among the 22 worst-response-associated lipids, 20 were triglycerides, 30% of total triglycerides (Figure 1E). Triglycerides with lower carbon number (40–48 carbons in fatty acid residues) were most affected (Figure 1F). These triglycerides were also among the least abundant ones (Figure S3).

**Figure 2:**
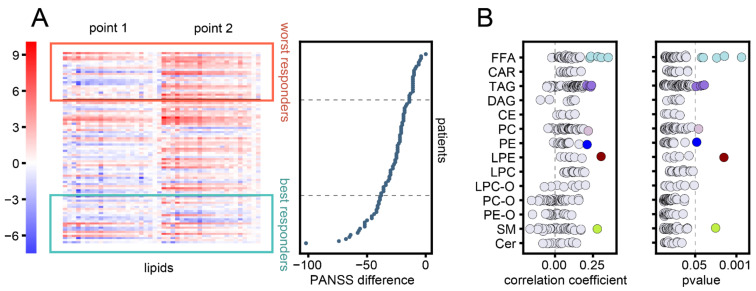
Association between lipid changes and symptom improvement. (**A**) The normalized base 2 log-transformed (log2) abundances of the 22 worst-response-associated lipids at first and second time points. For each lipid, the log2 abundances were normalized by the lipid mean value for all patients at the first time point. Individuals were sorted by PANSS difference between the two time points, plotted to the right. Colored boxes and grey horizontal lines delineate the best and worst responders. (**B**) Spearman correlation coefficients (left) and corresponding *p*-values (right) between changes in lipid abundances and differences in PANSS score. Lipids with nominally significant *p*-values (< 0.05) are marked in color. Dashed lines delineate *p*-value 0.05 and correlation coefficient 0.

The increase in worst-response-associated lipids was not restricted to 25% of patients displaying the least improvement in PANSS scores, as individuals in the interquartile range with less symptom improvement also displayed this increase in lipid abundance (Figure 2A). Correlation analysis between PANSS score reduction and lipid abundance changes yielded 13 nominally significant lipids, including free fatty acids and triglycerides (Spearman correlation, nominal *p* < 0.05) (Figure 2B), but these lipids did not pass the 5% threshold after correction for multiple testing (Benjamini–Hochberg correction).

### 3.3. Influence of Medication

**Figure 3:**
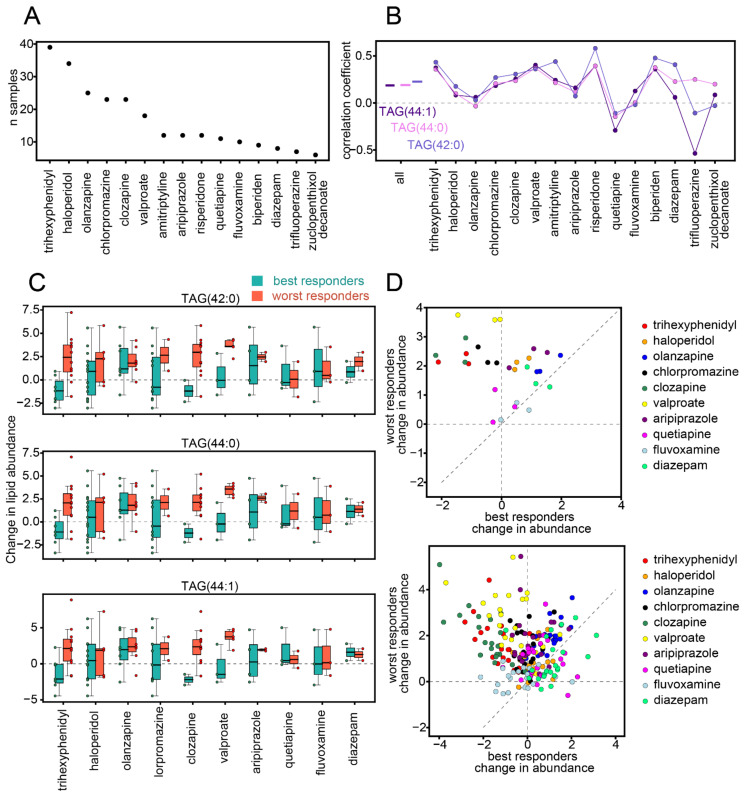
Medication effect. (**A**) Number of individuals receiving specific medications. The schema includes the medications that were administered to more than 5 individuals. (**B**) Spearman correlation coefficients between changes in PANSS scores and changes in lipid levels between the two time points, for all patients and only patients receiving a particular medication. Three worst-response-associated lipids with the strongest statistical effect (*p* < 0.0005 Wilcoxon signed-rank test for changes in worst responders) are shown: TAG 42:0, TAG 44:0, and TAG 44:1. Different color shades correspond to the three different lipids. The schema includes the medications that were administered to more than 5 individuals. (**C**) Changes in lipid abundances for best (green) and worst (orange) responders that were receiving a particular medication. Changes in abundances were calculated as the base 2 log-transformed fold-changes (log2FCs) between the time points. The particular triglyceride species are marked at the top. Medications that were administered to at least two worst and two best responders are shown. In addition to the boxplots, abundance changes for each individual are marked as a circle. Boxplot whiskers correspond to the standard boxplot definition. (**D**) Median changes in lipid abundances for best and worst responders that were receiving a particular medication, colored by medication. Top: TAG 42:0, TAG 44:0, and TAG 44:1. Bottom: all 22 worst-response-associated lipids. Changes in abundances were calculated as the log2FC between the time points. Medications that were administered to at least two worst and two best responders are shown.

Because the association between changes in lipid abundance levels and symptom severity could be confounded by medication regimens, we aimed to study this association confined to patients receiving the same drugs. Individuals enrolled in this study received a variety of different medications and often more than one medication at once. In total, 40 different medications were administered to at least one individual, and 15 medications were represented six or more times in the sample population (Figure 3A). We restricted the correlation analysis between symptom improvement and changes in lipid abundance levels to individuals receiving each of these 15 medications. For the three triglyceride species with the strongest worst-response-associated effect (*p* < 0.0005: TAG 42:0, TAG 44:0, and TAG 44:1), these correlations were positive for most of the medications (11, 13, and 13 out of the 15 medications for TAG 42:0, TAG 44:0, and TAG 44:1, respectively; Figure 3B), which is significantly more than expected by chance (binomial test *p* = 0.059, 0.0037, and 0.0037). For the other worst-response-associated lipids, the correlations were positive for 10–15 of the 15 medications, with 41% percent of lipids showing significant values and 45% borderline significant values (binomial test *p* < 0.05 and *p* = 0.059, respectively; Figure S4).

Similarly, we assessed the changes in lipid abundances between the two time points for best responders and worst responders while restricting the analysis to individuals receiving a particular medication. In total, there were 10 medications for which there were at least two best responders and two worst responders. For TAG 42:0, TAG 44:0, and TAG 44:1, not only were the median lipid abundances increased in worst responders for all of these 10 medications, but for most of them, median changes in worst responders were higher than median changes in best responders (9, 10, and 9 of the 10 medications for TAG 42:0, TAG 44:0, and TAG 44:1, respectively; binomial test *p* = 0.0107, 0.0001, and 0.0107; Figure 3C). For these three triglyceride species, quetiapine, fluvoxamine, and diazepam were least associated with symptom severity (Figure 3D). However, quetiapine, fluvoxamine, and diazepam were represented by the smallest number of individuals. Among the medications for which the difference between worst and best responders was strongest, we noted clozapine, chlorpromazine, valproate, and trihexyphenidyl (Figure 3D). For the rest of the worst-response-associated lipids, results were similar, except for haloperidol, for which changes in worst responders were similar to best responders for most of these lipids (Figure 3D).

### 3.4. Influence of Sex and Age

While age and time between the two plasma collection points were similar for best and worst responders (Figures S5 and S6), there was a strong sex imbalance. Most of the best responders were female (Figure S7), making sex a potential confounding factor for symptom improvement. Comparison of changes in female best and worst responders demonstrated that female worst responders had increased abundances for worst-response-associated lipids compared to best responders, as was the case for the best and worst responding individuals of the two sexes (Figure S8). For both female and male individuals, the correlation between PANSS score reduction and changes in lipid abundances for worst-response-associated lipids was positive for 100% and 82% of the lipids (binomial test; *p* = 2.38 × 10^−7^ and 0.0022; Figure S9), indicating that the observed effect of increased worst-response-associated lipids at the second time point could not be explained by the imbalance in sex distribution observed in the data.

Additionally, we aimed to investigate whether there was an interaction effect between PANSS score changes, sex, and age on changes in lipid abundances between the two time points using analysis of variance (ANOVA). Higher-order interactions between PANSS score changes, sex, and age (PANSS change * sex * age) did not show any detectable statistical effect (ANOVA, 1.28 fold excess of nominally significant lipids at *p* < 0.05, permutation *p =* 0.46; Figure S10A; Table S2). For PANSS scores changes and sex, as well as PANSS scores and age, there did seem to be a significant interaction effect, though not strong enough to pass the multiple testing correction (ANOVA, 3.8 and 3.9 fold excess of nominally significant lipids at *p* < 0.05, permutation *p =* 0.07 and *=* 0.08, respectively; Figure S10A; Table S2). The nominally significant lipids for the interaction effect PANSS change * sex (nominal *p* < 0.05) were particularly enriched with ether phosphatidylcholines (PC-O) (permutation *p* < 0.0001; Figure S10B), while the nominally significant lipids for interaction effect PANSS change * age (nominal *p* < 0.05) were enriched with both ether phosphatidylcholines and ether phosphatidylethanolamines (PC-O, PE-O), as well as sphingomyelins (SM) (permutation *p =* 0.0019, 0.017, 0.0158, respectively; Figure S10C).

### 3.5. Lipid Profiles at First Time Point

Unlike changes in lipid abundances between the two time points, the lipid abundances at the first time point could potentially function as actionable predictors of symptom improvement. However, blood plasma lipids are subject to interindividual variability related to various factors, including basic parameters like sex and age [30]. Like others [30], we have found that both sex and age have a strong effect on lipid abundances (number of significant lipids *n* = 41 and *n* = 36 for sex and age, respectively; ANOVA, Benjamini–Hochberg correction FDR 5%; Figure S11A; Table S2). We did not find statistically significant main effects or interaction effect on the lipid abundances related to the change in PANSS scores (ANOVA, no significant lipids after Benjamini–Hochberg correction FDR 5%; factors: PANSS change, PANSS change * sex, PANSS change * age; Table S2). However, the interaction effect between PANSS score changes and sex on the lipid abundances was stronger than the other interactions and was also stronger than the main effect of PANSS score changes on lipid abundances (ANOVA for PANSS change * sex: 2.45 fold excess of nominally significant lipids at *p* < 0.05, permutation *p =* 0.15; ANOVA for PANSS change, PANSS change * age, PANSS change * sex * age: 1.27, 0.17, 0.27 fold excess of nominally significant lipids at *p* < 0.05, permutation *p =* 0.45, 0.91, 0.91; Figure S11B; Table S2).

Symptom improvement after antipsychotic treatment was shown to be sex-dependent in this study (Figure S7) as well as others [37,38]. For this reason, the association between symptom improvement and lipid abundances at the first point was strongly confounded by sex, which warranted the separate investigation for males and females. Consistent with the stronger PANSS change * sex interaction effect in the analysis of variance, we observed that correlation coefficients between PANSS score changes and lipid abundances at the first time points were inconsistent between females and males (Figure S11C; Table S2). The correlation coefficients of the nominally significant (Spearman correlation nominal *p* < 0.05) lipids had mostly opposite signs for females and males. However, for both females and males, these correlation levels were not statistically significant after correction for multiple testing, and the observed effect would require further confirmations.

## 4. Discussion

In this study, we assessed the abundances of 322 lipids in the blood plasma of 92 patients diagnosed with schizophrenia collected at two points in time during the individuals’ inpatient treatment. We aimed to investigate the association between individual changes in lipid abundances and improvement in symptom severity. We found that, for patients with the least improvement in symptom severity, 22 lipids, including 20 triglyceride species, were increased at the second time point, while patients with most improvement did not demonstrate the same increase in lipid levels. The most affected triglycerides contained a lower number of carbons (40–48 carbons in fatty acid residues), while the triglyceride species most abundant in blood plasma were not among the significantly increased lipids.

Others have studied the association between lipid levels and symptom improvement. Levels of total triglycerides, which are more routinely used in clinical practice but provide a rougher approximation of the blood plasma lipidome, have been shown to correlate negatively with PANSS score differences in some studies [39]. This effect, however, was not reproduced by others [39]. In our study, we have found the opposite direction of correlation for triglyceride species. This effect, however, does directly contradict the former studies, since the abundances of triglyceride species can differ in orders of magnitude. Total triglyceride measurements are strongly represented by the top most abundant lipid species, which we did not find to be significantly associated with symptom improvement. In our study, the triglycerides associated with symptom improvement were triglycerides containing a lower number of carbons. Interestingly, one study has found that an increase in triglycerides with these chain length ranges was associated with weight gain in first psychotic episode patients after undergoing treatment [40]. In general, short-chain triglycerides, in particular, have been found to be associated with diabetes and nonalcoholic fatty liver disease [41,42]. These observations suggest that the increase in particular triglycerides we detected in worst responders could be related to metabolic abnormalities induced by antipsychotic treatment, affecting the treatment response of the patients.

Antipsychotic treatment has previously been shown to affect both clinical lipid measurements, such as total triglycerides, and compounds at the level of lipid species [12,25,32,43,44]. Some antipsychotics have been shown to have a larger effect on the blood plasma lipids than others, and their effect on particular compounds was different between them. Therefore, symptom-dependent imbalance in treatment regimen could potentially confound the association between lipid levels and treatment response. Nevertheless, the association between specific triglyceride levels and symptom improvement we report could not be explained by a bias in the treatment regimen, but rather the effect was reproduced for most of the medications independently. Indeed, for most of the medications, changes in lipid levels in patients receiving a particular medication were higher for worst responders compared to best responders. Among the medications for which the difference between worst and best responders was strongest, we noted clozapine (atypical antipsychotic), chlorpromazine (typical antipsychotic), valproate (mood stabilizer), and trihexyphenidyl (antimuscarinic). However, one limitation of the study was the large variety of medications administered to the patients, resulting in patients receiving multiple psychopharmacological drugs simultaneously, and some medications were represented by only a small number of patients.

Consistent with reported differences in treatment response between sexes [37,38], we saw that females demonstrated more improvement in symptom severity after inpatient treatment than males. Nevertheless, the symptom-dependent increase in worst-response-associated lipids was present for females and males alike. However, the number of males with larger symptom improvement was relatively small, and a significant sex-dependent association could be present but not detected in our analysis. We did find an enrichment of ether phosphatidylcholines (PC-O) among the nominally significant lipids for the interaction effect between changes in PANSS scores and sex on the changes in lipid abundances. However, the differing ranges of symptom improvement for females and males call for a more detailed investigation of the potential sex-specific effects, possibly with larger sample sizes.

While lipid abundances at the first time point could have been considered as predictors of future symptom improvement, we did not find a significant association between these abundances and the PANSS score changes. We did find that these associations were different for females and males, with the respective correlations showing opposite directions. However, these effects were not particularly strong and were not statistically significant after correction for multiple testing, warranting further investigation.

While there is ample evidence of metabolic abnormalities linked to schizophrenia and other psychiatric disorders, such as diabetes, cardiovascular disorder, and weight gain [45,46,47,48,49], it is still unclear whether these abnormalities co-occur with schizophrenia because of side effects from medications, lifestyle and diet, certain genetic predispositions, or possibly negative physiological impact of the psychiatric disorder. In this study, we have likewise found a metabolic signature associated with the severity of schizophrenia manifestation, which could also be linked to metabolic alterations found in common metabolic disorders. We have also found some weaker evidence for possible differences in the association of symptom improvement and lipid response between females and males. More studies are needed to recognize the implications of such findings for the treatment and understanding of the pathophysiology of schizophrenia.

## Data Availability

The data are available from the corresponding author upon reasonable request.

## Supplementary Materials

The following are available online at https://www.mdpi.com/article/10.3390/biom11050720/s1, Figures S1–S11, Table S1: sample information, Tables S1 and S2: lipid annotation and statistical results.

## Abbreviations

DAG	diacylglycerol
TAG	triacylglycerol
FFA	free fatty acid
CAR	acylcarnitine
CE	cholesteryl ester
PC	phosphatidylcholine
PC-O	plasmanyl-/plasmenyl-phosphatidylcholine
LPC	lysophosphatidylcholine
LPC-O	lyso- plasmanyl-/plasmenyl- phosphatidylcholine
PE	phosphatidylethanolamine
PE-O	plasmanyl-/plasmenyl- phosphatidylethanolamine
LPE	lysophosphatidylethanolamine
Cer	ceramide
SM	sphingomyelin
