# Supplementary material for: Shorter Chain Triglycerides Are Negatively Associated with Symptom Improvement in Schizophrenia
Source: Biomolecules. 2021 May 11;11(5):720. doi: 10.3390/biom11050720 (PMC8151512; doi:10.3390/biom11050720)
Supplement: Supplementary file 1 [file biomolecules-11-00720-s001.zip › suppl/PANSSsuppl_fv.pdf]

Shorter chain triglycerides associated with less symptom improvement in schizophrenia.

Anna Tkachev [1,*], Elena Stekolshchikova [1], Nickolay Anikanov [1], Svetlana Zozulya [2], Aleksandra Barkhatova [2], Tatiana Klyushnik [2], Daria Petrova [1]

[1] V. Zelman Center for Neurobiology and Brain Restoration, Skolkovo Institute of Science and Technology, Moscow, 121205, Russia
[2] Mental Health Research Center, Moscow, 115522, Russia

* Correspondence: anna.tkachev@skolkovotech.ru

Supplementary Figures

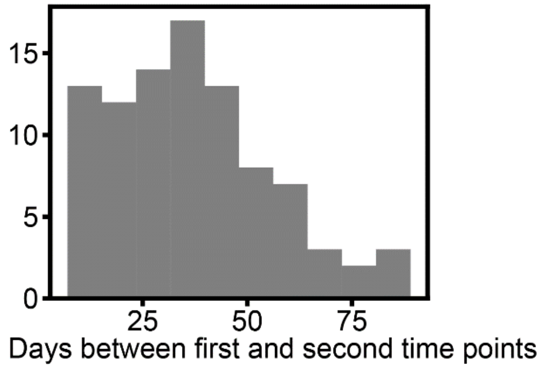

Supplementary Figure 1.

Distribution of time between first and second time points at which the plasma samples were collected.

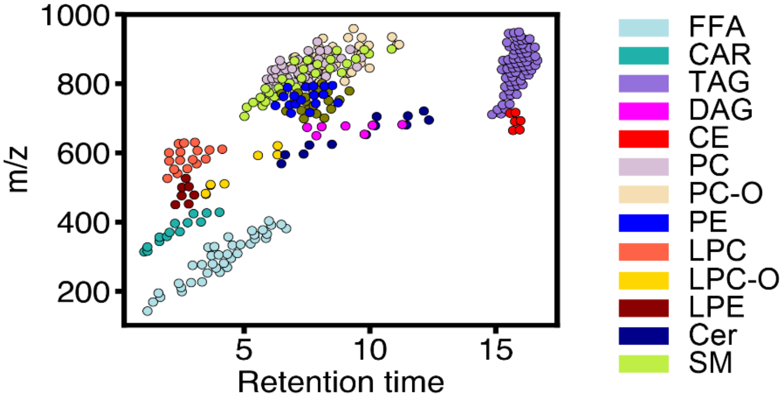

Supplementary Figure 2.

Retention times and mass-to-charge (m/z) values for the annotated lipids. Lipids are colored by lipid class, marked to the right.

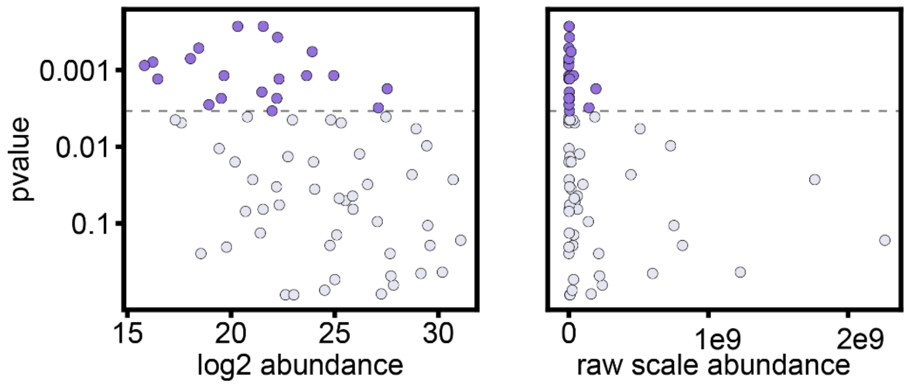

Supplementary Figure 3.

Relationship between Wilcoxon paired test p-values for worst responders and the average lipid abundances of triglyceride lipid species. Worst-response associated triglycerides are marked in purple, the other triglycerides are marked in gray. Dashed line delineates 5% false-discovery-rate cutoff after correction for multiple testing. Base two log-transformed abundances are visualized on the left, raw-scale abundances on the right.

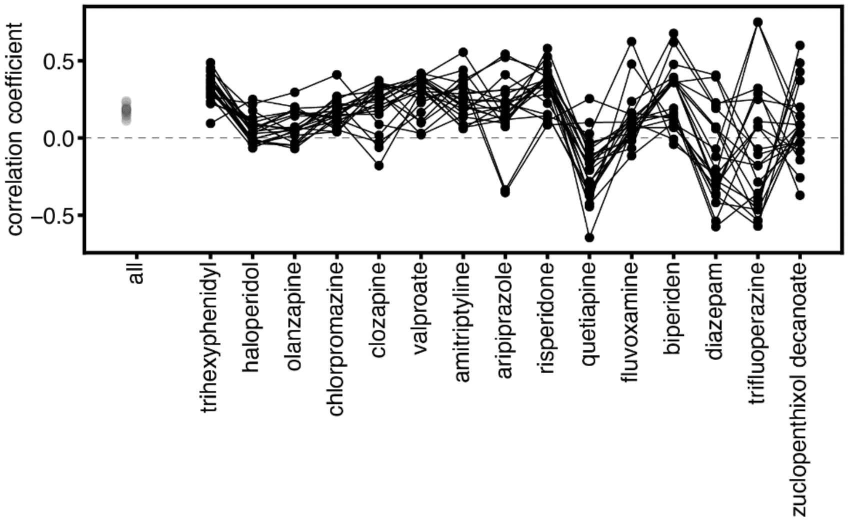

Supplementary Figure 4.

Spearman correlation coefficients between changes in PANSS scores and changes in lipid levels between the two time points, for all patients and only patients receiving a particular medication. All worst-response-associated lipids are shown. The schema includes the medications that were administered to more than 5 individuals.

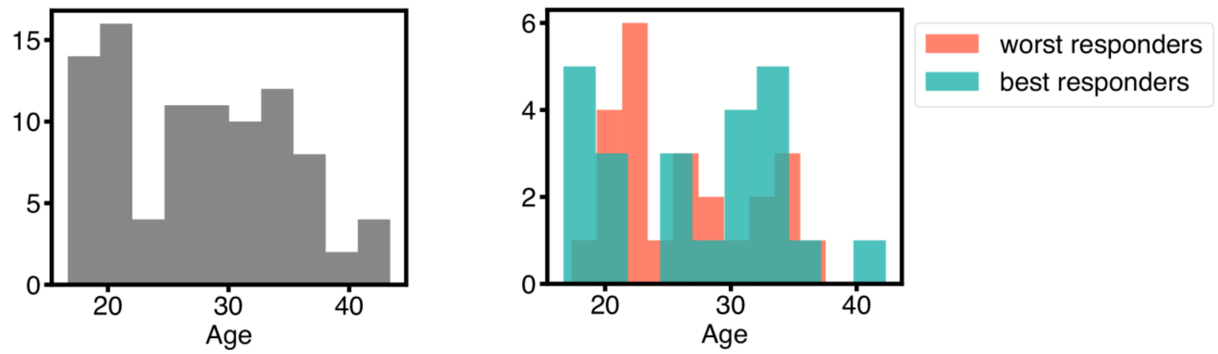

Supplementary Figure 5.

Distribution of ages for all individuals (left) and worst and best responders (right).

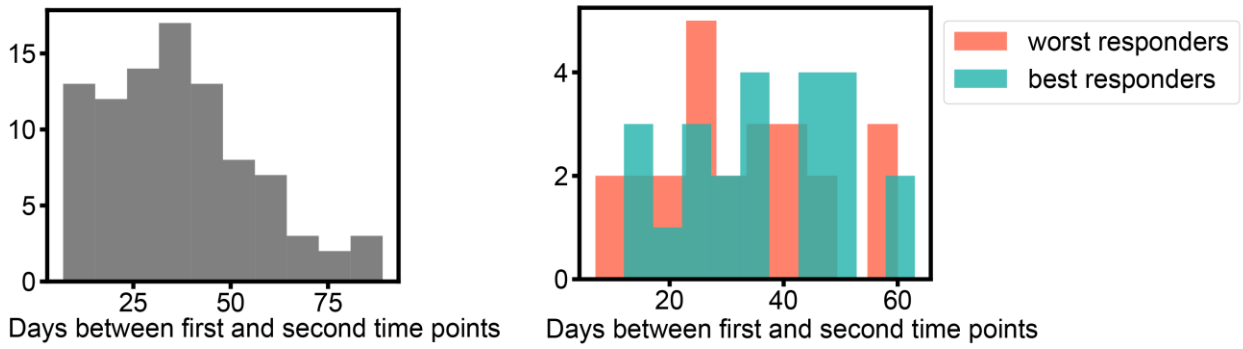

Supplementary Figure 6.

Distribution of time between first and second time points at which the plasma samples were collected. Left: all individuals. Right: best and worst responders.

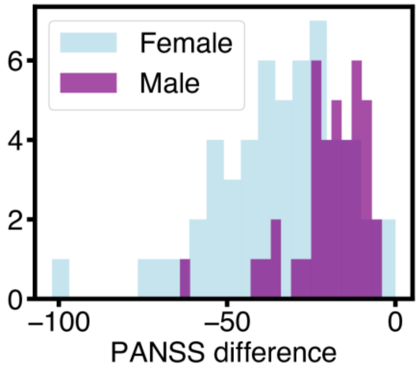

Supplementary Figure 7.

Distribution of differences in PANSS scores between the two time points for female and male individuals

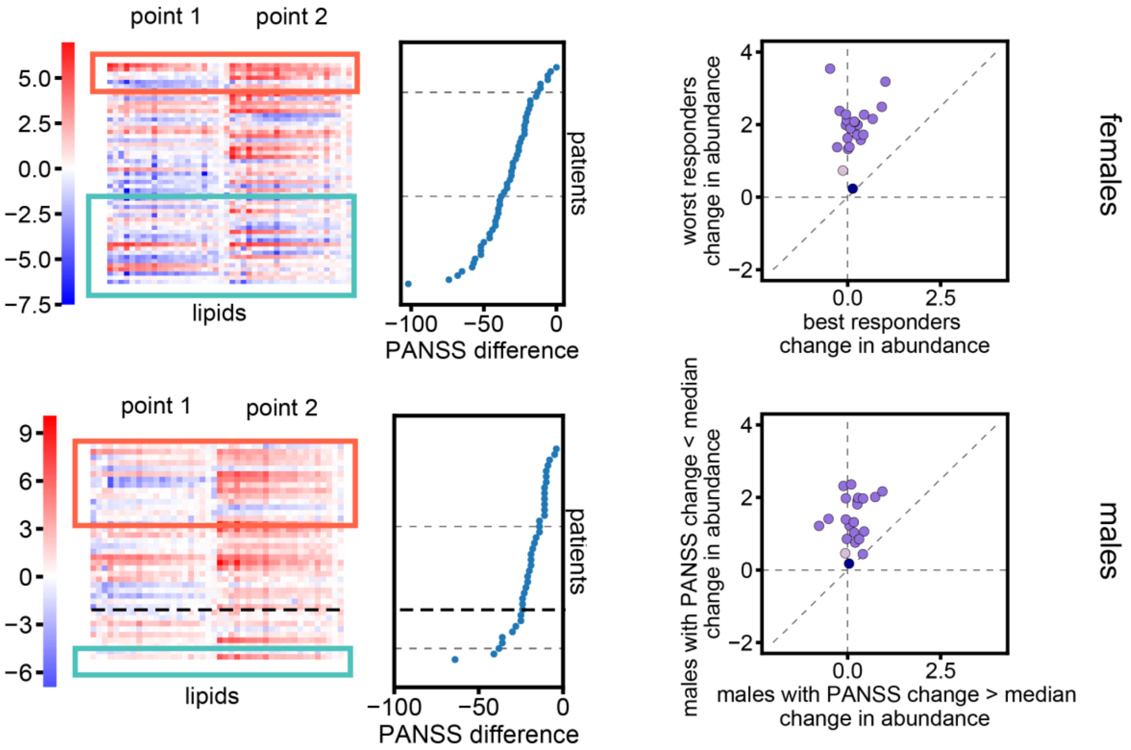

Supplementary Figure 8. The association between changes in PANSS score and the abundances of worst-response-associated lipids at first and second time point, for females and males separately.

(top left): For female individuals, the normalized base two log-transformed (log2) abundances of the worst-response-associated lipids at first and second time point. For each lipid, the log2 abundances were normalized by the lipid mean value for all patients at the first time point. The female individuals were sorted by PANSS difference between the two time points, plotted to the right. Colored boxes and dashed grey horizontal lines delineate the best and worst responders. (top right): Median changes in lipid abundances between the two time points for best and worst female responders. All worst-response-associated lipid are shown, colored by lipid class, TAG (purple), PC (dusty rose), Cer (dark blue). Changes in abundances were calculated as the log2 fold-changes between the time points.

(bottom left): For male individuals, the normalized base two log-transformed (log2) abundances of the worst-response-associated lipids at first and second time point. For each lipid, the log2 abundances were normalized by the lipid mean value for all patients at the first time point. The male individuals were sorted by PANSS difference between the two time points, plotted to the right. Colored boxes and dashed grey horizontal lines delineate the best and worst responders. Black dashed horizontal line delineates the median change in PANSS score for all samples. (bottom right): Median changes in lipid abundances between the two time points for males with PANSS changes less than and greater than the median. All worst-response-associated lipid are shown, colored by lipid class, TAG (purple), PC (dusty rose), Cer (dark blue). Changes in abundances were calculated as the log2 fold-changes between the time points.

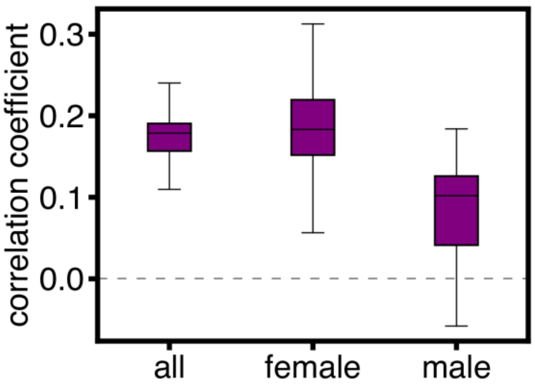

Supplementary Figure 9.

The Spearman correlation coefficients between changes in PANSS scores and the changes in abundances of the worst-response-associated lipids. Left to right: all individuals, only female individuals, only male individuals.
